# Assessment of Tyrosine Kinase Inhibitors and Survival and Cardiovascular Outcomes of Patients With Non–Small Cell Lung Cancer in Taiwan

**DOI:** 10.1001/jamanetworkopen.2023.13824

**Published:** 2023-05-17

**Authors:** Wei-Ting Chang, Hui-Wen Lin, Ting-Chia Chang, Sheng-Hsiang Lin, Yi-Heng Li

**Affiliations:** 1 Institute of Clinical Medicine, College of Medicine, National Cheng Kung University, Tainan, Taiwan; 2 Division of Cardiology, Department of Internal Medicine, Chi-Mei Medical Center, Tainan, Taiwan; 3 Department of Biotechnology, Southern Taiwan University of Science and Technology, Tainan, Taiwan; 4 Department of Internal Medicine, National Cheng Kung University Hospital, College of Medicine, National Cheng Kung University, Tainan, Taiwan; 5 Biostatistics Consulting Center, National Cheng Kung University Hospital, College of Medicine, National Cheng Kung University, Tainan, Taiwan; 6 Division of Pulmonology, Department of Internal Medicine, Chi Mei Medical Center, Tainan, Taiwan; 7 Department of Public Health, College of Medicine, National Cheng Kung University, Tainan, Taiwan

## Abstract

**Importance:**

Tyrosine kinase inhibitors (TKIs) have been recognized as the standard treatment for patients with non–small cell lung cancers (NSCLCs) and epidermal growth factor receptor (EGFR) sequence variation. Although TKIs have been reported to cause cardiotoxicity, they are widely administered owing to the high prevalence of *EGFR* sequence variation in Taiwan.

**Objective:**

To compare the outcomes of death and major adverse cardiac and cerebrovascular events among patients with NSCLC who use and do not use TKIs in a national cohort.

**Design, Setting, and Participants:**

Using data from the Taiwanese National Health Insurance Research Database and National Cancer Registry, patients treated for NSCLC from 2011 to 2018 were identified, and their outcomes were analyzed, including death and major adverse cardiac and cerebrovascular events (MACCEs; such as heart failure, acute myocardial infarction, and ischemic stroke) after adjusting for age, sex, cancer stage, comorbidities, anticancer therapies, and cardiovascular drugs. The median follow-up duration was 1.45 years. The analyses were performed from September 2022 to March 2023.

**Exposures:**

TKIs.

**Main Outcomes and Measures:**

Cox proportional hazards models were used to estimate death and MACCEs in patients treated with and without TKIs. Given that death may reduce the incidence of cardiovascular events, the competing risk method was used to calculate the MACCE risk after adjustment for all potential confounders.

**Results:**

Overall, 24 129 patients treated with TKIs were matched with 24 129 patients who did not receive TKIs (24 215 [50.18%] were female; and the mean [SD] age was 66.93 [12.37] years). Compared with those not receiving TKIs, the TKI group presented with a significantly lower hazard ratio (HR) of all-cause death (adjusted HR, 0.76; 95% CI, 0.75-0.78; *P* < .001), and the reason for death was primarily cancer. In contrast, the HR of MACCEs significantly increased (subdistribution HR, 1.22; 95% CI, 1.16-1.29; *P* < .001) in the TKI group. Furthermore, afatinib use was associated with a significantly reduced risk of death among patients receiving various TKIs (adjusted HR, 0.90; 95% CI, 0.85-0.94; *P* < .001) compared with those receiving erlotinib and gefitinib, although the outcomes of MACCEs were similar between the 2 groups.

**Conclusions and Relevance:**

In this cohort study of patients with NSCLC, TKI use was associated with reduced HRs of cancer-related death but increased HRs of MACCEs. These findings suggest the importance of close monitoring of cardiovascular problems in individuals receiving TKIs.

## Key Points

**Question:**

What are the incidence and outcomes of mortality and major adverse cardiac and cerebrovascular events (MACCEs) among patients diagnosed with non–small cell lung cancer (NSCLC) receiving tyrosine kinase inhibitors (TKIs) compared with those not receiving TKIs?

**Findings:**

In this nationwide cohort study of 24 129 patients with NSCLC who received TKIs and 24 129 matched controls who did not receive TKIs in Taiwan, although TKI use was associated with a lower probability of mortality in patients with NSCLC, the outcomes of MACCEs significantly increased among patients receiving TKI vs those who were not.

**Meaning:**

This study’s findings suggest the importance of close monitoring of cardiovascular problems for TKI use in patients with NSCLC.

## Teaser

This cohort study compares risk of death and major adverse cardiac and cerebrovascular events among patients with non–small cell lung cancer (NSCLC) receiving tyrosine kinase inhibitors (TKIs) vs those not receiving TKIs in a nationwide cohort in Taiwan.

## Introduction

Lung cancer causes more deaths than any other cancer in both men and women worldwide^1,2^; non–small cell lung cancer (NSCLC) accounts for approximately 85% of lung cancers, and approximately 50% of patients with newly diagnosed NSCLC are in metastatic status.^1,2^ Although compared with supportive care, platinum-based systemic chemotherapy has been found to improve symptoms and patient survival, overall survival in patients with advanced NSCLC remains low, and the 5-year survival is only 3.5%.^3,4^ With a better understanding of the role of tyrosine kinases in signal transduction and proliferation and differentiation in lung cancer cells, tyrosine kinase inhibitors (TKIs) have dramatically increased the survival of patients with epidermal growth factor receptor (*EGFR*) sequence variation–positive NSCLC.^5,6^ In addition, the presence of an *EGFR* sequence variation is not only prognostic for better overall survival but predictive of response to TKIs.^7^ Lin et al^8^ reported that the 5-year survival among patients with *EGFR*–sequence variant metastatic NSCLC increased to 14.6%. In particular, Shi et al^9^ reported *EGFR*–sequence variation prevalence of up to 50% in patients in East Asia compared with 12.8% in Europe in the PIONEER project, which is a global epidemiological study focusing on *EGFR* sequence variations in patients with newly diagnosed advanced NSCLC in Asia. Furthermore, in a meta-analysis, Zhang et al^10^ reported an overall prevalence for *EGFR* sequence variations of 32.3%, ranging from 38.4% in China to 14.1% in Europe. This highlighted a potential issue associated with the widespread prescribing of TKIs in Asia.

However, cardiac dysfunction induced by anticancer drugs adversely affects the survival and quality of life of patients with NSCLC.^11,12^ TKIs has been reported to exacerbate myocardial ischemia, heart failure, fatal arrhythmia, and hypertension^13,14,15,16^ but evidence on the long-term effects of TKIs on survival and cardiovascular outcomes in patients with NSCLC is insufficient. Therefore, using a national cohort, we compared the risks of death and adverse cardiovascular outcomes in patients with NSCLC receiving TKIs vs those not receiving TKIs. In addition, we examined all-cause mortality and major adverse cardiac and cerebrovascular events (MACCEs) in patients receiving erlotinib, afatinib, or gefitinib to investigate whether there are differences in hazards between first- and second-generation TKIs.

## Methods

### Patients and Study Design

Using the Taiwanese National Health Insurance Research Database (NHIRD) and National Cancer Registry, in this retrospective cohort study we identified patients with newly diagnosed NSCLC from 2011 to 2018. According to the insurance regulation in Taiwan, the first-line TKIs were only reimbursed for advanced (stage IIIb, IIIc, or IV) lung adenocarcinoma harboring *EGFR* sequence variation, and restricted for monotherapy.^17^ The cancer stage recorded by National Cancer Registry was the date of the diagnosis of NSCLC. Patients with a history of NSCLC, those aged younger than 18 years, those with incomplete data, and those who received TKIs in the past year before the index date were excluded from the study; medical records after death were also excluded. The first day of TKI use was set as the index date in the present study. The control group included patients who received no TKI treatment throughout the study period and whose propensity score was matched 1:1 with patients who received TKI treatment more than 90% of the time in the subsequent 30 days after enrollment. The data used in the present study were obtained from the original claims database for reimbursement of all Taiwanese residents of the NHIRD.^18,19^ The accuracy of NHIRD has been validated in previous studies.^18,19^ Details of patients’ age, sex, medical history, concomitant drug use within the last 3 months, and treatments or procedures were obtained from this database. Before 2015, the diagnostic codes in NHIRD were identified using the *International Classification of Diseases, Ninth Revision, Clinical Modification (ICD-9-CM)*, whereas after 2016, they were identified using the *International Statistical Classification of Diseases, Tenth Revision, Clinical Modification (ICD-10-CM)*. Notably, using NHIRD, the continuous claim data for the same patient could be tracked. The *ICD-9-CM* and *ICD-10-CM* diagnosis and treatment codes are presented in eTable 1 in Supplement 1. The flowchart of the present study is shown in the eFigure in Supplement 1.

The National Cheng Kung University Hospital institutional review board approved this study, and because this was a retrospective study, the requirement for an informed consent form was waived. We followed the Strengthening the Reporting of Observational Studies in Epidemiology (STROBE) reporting guideline.

### Study End Point

The primary end point was death, which was classified into 3 categories based on causes: cardiovascular disease, cancer, and other causes. New onset heart failure (HF), acute myocardial infarction (AMI), and ischemic stroke following TKI use in patients with NSCLC were identified as the secondary end point. All patients were followed up from the index date to the date of death or the date when they were lost to follow-up. Given that *ICD-9-CM* was replaced by *ICD-10-CM* by Taiwan National Health Insurance in 2016, both *ICD-9-CM* and *ICD-10-CM* codes (eTable 1 in Supplement 1) were used to identify end points in the primary outcome during follow-up. The analysis were performed from September, 2022 to March 2023. The median follow-up time was 1.45 years, and the last follow-up appointment was at the end of 2020.

### Statistical Analysis

Continuous variables are represented as means with SDs, and categorical variables are represented as numbers and percentages. Furthermore, despite the nonrandomized nature of the study, propensity score analysis was performed to reduce any selection bias caused by differences in clinical characteristics between the groups.^20^ The propensity score is defined as the probability of being exposed to treatment based on a study participant’s baseline characteristics. In the present study, the propensity score for the use or nonuse of TKIs was calculated using multivariate logistic regression analysis based on the factors of index year, age, sex, cancer stage, procedures used, drugs used, and comorbidities before admission. Furthermore, instead of using statistical tests, the absolute standardized mean difference (ASMD) was used to compare the distributions of clinical features between the 2 groups. An ASMD of 0.1 indicated an insignificant difference between the 2 groups. ASMD was calculated as the mean or proportion of a variable divided by the pooled estimate of the standard deviation of that variable. Subsequently, using a multivariate Cox proportional hazards model, we analyzed the association between the end points and different treatments. Furthermore, all these potential confounders were taken into account when calculating the hazard ratios (HRs) and their 95% CIs from the Cox models. In addition, given that death may reduce the incidence of cardiovascular events, the competing risk method (subdistribution HR) was used to calculate the HRs of MACCE from the Cox regression model after adjustment for all potential confounders. Moreover, the difference between the groups was compared using the Gray test. Results were plotted using the cumulative incidence function for outcome event and competing risk events of death. Furthermore, to estimate the *P* values for interactions in the subgroup analysis, we used the same Cox proportional hazards model (competing risk technique). The statistical significance was considered as 2-sided *P* < .05. All data analyses were performed using SAS 9.4 for Windows (SAS Institute) from September 2022 to March 2023.

## Results

Among the 48 258 patients treated for NSCLC with or without TKIs, 24 215 (50.18%) were female; and the mean (SD) age was 66.93 (12.37) years. There were 24 129 patients diagnosed with NSCLC and treated with TKIs, and they were matched with 24 129 patients diagnosed with NSCLC who did not receive TKIs. The age and sex of these 2 groups were comparable (Table 1). Notably, 21 854 patients (45.29%) had a history of hypertension, whereas 10 458 patients (21.67%) had diabetes, 10 535 (21.83%) had hyperlipidemia, and 9741 (20.19%) had chronic obstructive pulmonary disease. Overall, 13 242 (27.44%) of the enrolled patients received ACEIs/ARBs, 10089 (20.91%) received β-blockers, and 7731 (16.02%) received antiplatelet drugs. Details of cancer treatments are provided in eTable 2 in Supplement 1. Notably, approximately 10% of anticancer drugs contain platinum analogs, such as cisplatin and carboplatin, in addition to TKIs.

**Table 1.** Baseline Characteristics of Patients With NSCLC Treated With or Without TKIs After Propensity Score Matching

Characteristics	Patients, No. (%)						ASMD
	Total (n = 48 258)		TKI use (n = 24 129)		No TKI use (n = 24 129)		
Age, y							
Mean (SD)	66.93 (12.37)		66.91 (11.92)		66.95 (12.80)		0.003
Median (IQR)	67.00 (18.00)		67.00 (17.00)		67.00 (19.00)		
Sex							
Male	24 043 (49.82)		11 351 (47.04)		12 692 (52.60)		0.11
Female	24 215 (50.18)		12 778 (52.96)		11 437 (47.40)		
Therapies for cancer							
Radiotherapy	1438 (2.98)		738 (3.06)		700 (2.90)		0.009
Operations (lobectomy)	2426 (5.03)		1078 (4.47)		1348 (5.59)		0.05
Anticancer drugs^a^	10 707 (22.19)		5653 (23.43)		5054 (20.95)		0.06
Platinum analogues	5219 (10.81)		2600 (10.78)		2619 (10.85)		0.003
Cardiovascular medications							
ACEIs/ARBs	13 242 (27.44)		6537 (27.09)		6705 (27.79)		0.02
β-blockers	10 089 (20.91)		4982 (20.65)		5107 (21.17)		0.01
Anti-platelet agents	7731 (16.02)		3812 (15.80)		3919 (16.24)		0.01
Anti-coagulants	1382 (2.86)		696 (2.88)		686 (2.84)		0.003
Statins	7440 (15.42)		3621 (15.01)		3819 (15.83)		0.02
Digoxin	685 (1.42)		348 (1.44)		337 (1.40)		0.004
MRA	1798 (3.73)		908 (3.76)		890 (3.69)		0.004
Antiarrhythmia drugs^a^	1170 (2.42)		583 (2.42)		587 (2.43)		0.001
Comorbidities							
Coronary artery disease	5812 (12.04)		2885 (11.96)		2927 (12.13)		0.005
Peripheral artery disease	926 (1.92)		469 (1.94)		457 (1.89)		0.004
Hypertension	21 854 (45.29)		10 837 (44.91)		11 017 (45.66)		0.02
Diabetes mellitus	10 458 (21.67)		5168 (21.42)		5290 (21.92)		0.01
Hyperlipidemia	10 535 (21.83)		5175 (21.45)		5360 (22.21)		0.02
Valve diseases	1336 (2.77)		674 (2.79)		662 (2.74)		0.003
Chronic obstructive pulmonary disease	9741 (20.19)		4721 (19.57)		5020 (20.80)		0.03
Asthma	3717 (7.70)		1849 (7.66)		18 68 (7.74)		0.003
Chronic kidney disease	4171 (8.64)		2001 (8.29)		2170 (8.99)		0.03
End-stage kidney disease	64 (0.13)		32 (0.13)		32 (0.13)		0.000

Abbreviations: ASMD, absolute standardized mean difference; ACEI/ARB, angiotensin-converting enzyme inhibitor/angiotensin receptor blocker; MRA, mineralocorticoid-receptor antagonists; NSCLC, non–small cell lung cancer; TKIs, tyrosine kinase inhibitors.

^a^ Anticancer drugs and antiarrhythmia drugs were listed in eTable 1 in Supplement 1.

### Risk of Death and Cardiovascular Outcomes Among Patients With NSCLC Receiving TKIs vs Not Receiving TKIs

**Figure 1.**
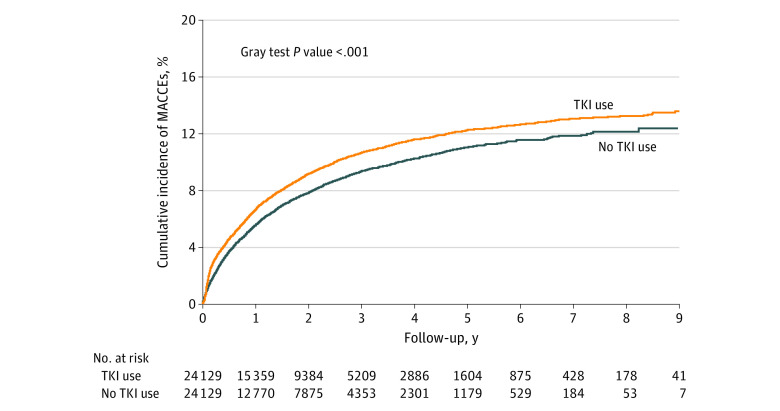
Accumulating Incidences of Major Adverse Cardiac and Cerebrovascular Events (MACCEs) Between Patients With Non–Small Cell Lung Cancers (NSCLC) Receiving Tyrosine Kinase Inhibitors (TKIs) vs Not Receiving TKIs

During the mean (SD) follow-up period of 1.92 (1.74) years, 37 796 (78.32%) of the studied population died (eTable 3 in Supplement 1). Patients receiving TKIs had a significantly lower risk of dying from any cause compared with those not receiving TKIs (crude HR, 0.90; 95% CI, 0.88-0.92; *P* < .001) (eTable 4 in Supplement 1). The association of TKI use with a lower overall death rate persisted after adjustment for age, sex, cancer stage, comorbidities, cancer therapy, and cardiovascular drugs (adjusted HR, 0.76; 95% CI, 0.75-0.78; *P* < .001) (eTable 4 in Supplement 1). Notably, the cause of death was primarily cancer (eTable 5 in Supplement 1). We compared the HRs of subsequent MACCEs, such as HF, AMI, and ischemic stroke, between the TKI group and non-TKI group during the follow-up period to further examine the association of TKIs with cardiovascular outcomes. Given that the majority of patients could die due to cancer, before they reached the cardiovascular end point, we adjusted mortality as a competing risk and observed a significant increase in MACCEs in those receiving TKI compared with those who did not (adjusted subdistribution HR, 1.22; 95% CI, 1.16-1.29; *P* < .001) (Table 2). The cumulative incidence of MACCEs among the TKI group was noticeably higher than that among the non-TKI group, as depicted in Figure 1. Additionally, the likelihood of HF (adjusted subdistribution HR, 1.10; 95% CI, 1.02-1.19; *P* = .02), AMI (adjusted subdistribution HR, 1.27; 95% CI, 1.06-1.51; *P* = .008), and ischemic stroke (adjusted subdistribution HR, 1.34; 95% CI, 1.24-1.44; *P* < .001) were significantly higher among the TKI group compared with the non-TKI group (Table 2).

**Table 2.** Event Number, Crude, and Adjusted sHR of Patients With NSCLC Treated With or Without TKIs

Variable	Patients, No (%)			Crude sHR (95% CI)	*P* value	Adjusted sHR (95%CI)^a^	*P* value
	Total (n = 48 258)	TKI use (n = 24 129)	No TKI use (n = 24 129) [Reference]				
All-cause death	37 796 (78.32)	19 338 (80.14)	18 458 (76.50)	NA	NA	NA	NA
MACCEs	5278 (10.94)	2848 (11.80)	2430 (10.07)	1.17 (1.11-1.23)	<.001	1.22 (1.16-1.29)	<.001
Heart failure	2443 (5.06)	1262 (5.23)	1181 (4.89)	1.06 (0.98-1.14)	.18	1.10 (1.02-1.19)	.02
Acute myocardial Infarction	511 (1.06)	283 (1.17)	228 (0.94)	1.23 (1.03-1.46)	.02	1.27 (1.06-1.51)	.008
Ischemic stroke	2822 (5.85)	1596 (6.61)	1226 (5.08)	1.29 (1.20-1.39)	<.001	1.34 (1.24-1.44)	<.001

Abbreviations: MACCEs, major adverse cardiac and cerebrovascular events; NSCLC, non–small cell lung cancer; TKIs, tyrosine kinase inhibitors; sHR, subdistribution hazard ratio.

^a^ Model was adjusted for age, sex, stage, therapies used during (radiotherapy, operation, antiarrhythmia drugs, anticancer drugs, platinum analogues), cardiovascular medication (angiotensin-converting enzyme inhibitor/angiotensin receptor blocker, β-blocker, anti-platelet agents, anticoagulants, statins, digoxin, mineralocorticoid-receptor antagonists), comorbidities (coronary artery disease, peripheral artery disease, hypertension, diabetes, hyperlipidemia, valve disease, chronic obstructive lung disease, asthma, chronic kidney disease, end-stage kidney disease).

### Subgroup Analysis of MACCEs Among Patients With vs Without TKI Use

**Figure 2.**
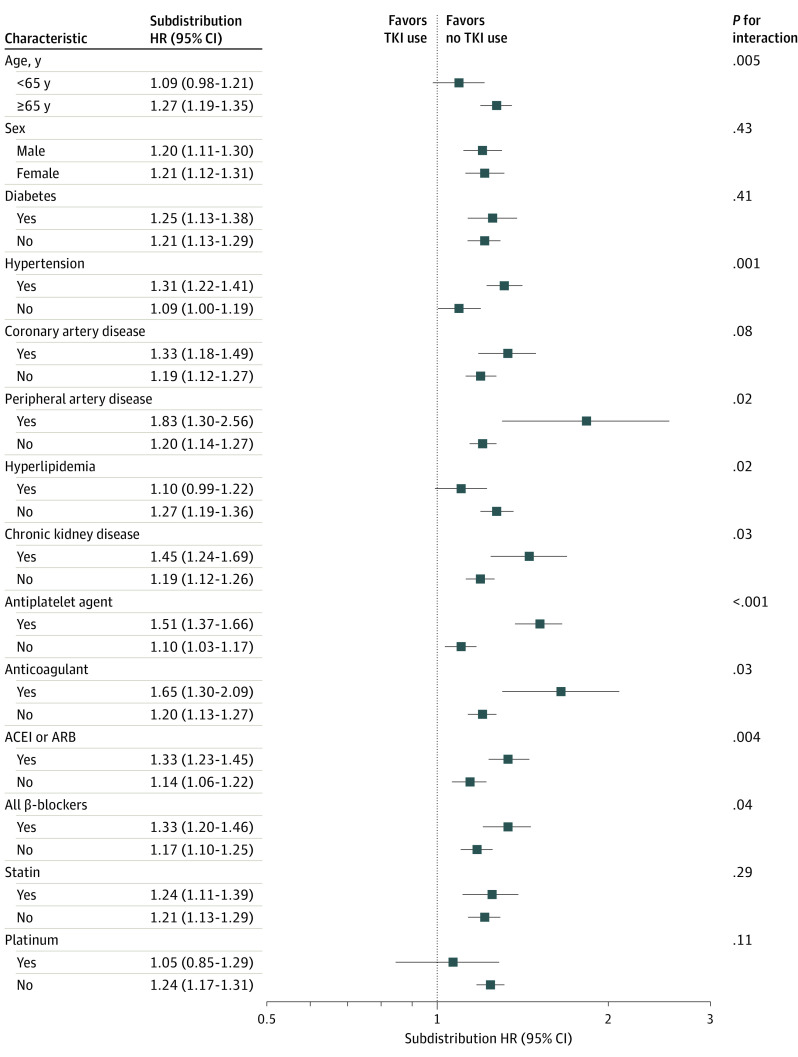
Subgroup Analysis of Major Adverse Cardiac and Cerebrovascular Events (MACCEs) Between Patients With Non–Small Cell Lung Cancers (NSCLC) Receiving vs Not Receiving Tyrosine Kinase Inhibitors (TKIs)

Based on our finding that the TKI group had higher HRs of MACCEs than the non-TKI group, we further aimed to identify if this phenomenon was observed in patients with other characteristics. In the subgroup analysis, we found that the probability of MACCEs increased in the TKI group, regardless of sex; history of diabetes, coronary artery disease, peripheral artery disease, or chronic kidney disease; or use of cardiovascular medications, whereas the risk increased in relatively younger patients (aged younger than 65 years) and in those without hypertension (Figure 2). In particular, the likelihood of MACCEs in patients receiving platinum therapy was negligible, but it steadily increased in patients not receiving platinum therapy.

### Comparison Between Different TKIs Regarding Death and Cardiovascular Outcomes

To further analyze whether the associations of TKIs with death and cardiovascular outcomes vary, we examined an additional cohort of 12 666 patients who received erlotinib, afatinib, or gefitinib with a matching of 1:1:1 (eTable 6 in Supplement 1). There were no discernible differences in age, sex, morbidity, cancer treatments, or cardiovascular medications between the 3 groups. Notably, patients receiving afatinib had a significantly lower probability of death (adjusted HR, 0.90; 95% CI, 0.85-0.94; *P* < .001) compared with those receiving erlotinib and getinib (Table 3). Moreover, compared with those receiving gefitinib, the hazards were increased for those receiving erlotinib (adjusted HR, 1.12; 95% CI, 1.07-1.18; *P* < .001), but they were decreased for those receiving afatinib (adjusted HR, 0.90; 95% CI, 0.85-0.94; *P* < .001). In contrast, the HRs of MACCEs were not different among the 3 groups. Given that 7889 patients (62.28%) receiving TKI died during the follow-up period, we adjusted mortality as a competing risk. However, compared with the HRs of MACCEs in patients receiving gefitinib, the HRs were similar among those receiving erlotinib (adjusted subdistribution HR, 0.98; 95% CI, 0.86-1.10, *P* = .70) and those receiving afatinib (adjusted subdistribution HR, 1.01; 95% CI, 0.89-1.15; *P* = .83) in the TKI group (eTable 7 in Supplement 1). Regarding the cause of the high mortality among those receiving TKI, the majority of patients died due to cancer-related causes, with the percentage being comparable in patients receiving erlotinib, gefitinib, or afatinib (eTable 8 in Supplement 1).

**Table 3.** Crude and Adjusted HRs of Patients With Lung Cancer Treated With Different TKIs, Including Erlotinib, Gefitinib, and Afatinib

Variable	Patients, No. (%)					Crude HR (95% CI)	*P* value	Adjusted HR (95% CI)^a^	*P* value
	Total (n = 12 666)	Gefitinib use (n = 4222) [Reference]	Erlotinib use (n = 4222)	Afatinib use (n = 4222)					
All cause death	8744 (69.04)	2916 (69.07)	3065 (72.60)	2763 (65.44)	Erlotinib	1.12 (1.07-1.18)	<.001	1.12 (1.07-1.18)	<.001
					Afatinib	0.86 (0.82-0.91)	<.001	0.90 (0.85-0.94)	<.001
MACCEs	1524 (12.03)	552 (13.07)	502 (11.80)	470 (11.13)	Erlotinib	0.96 (0.85-1.08)	.46	1.03 (0.91-1.16)	.68
					Afatinib	0.78 (0.69-0.88)	<.001	0.97 (0.85-1.10)	.58
Heart failure	665 (5.25)	249 (5.90)	220 (5.21)	196 (4.64)	Erlotinib	0.93 (0.77-1.11)	.41	1.03 (0.86-1.24)	.72
					Afatinib	0.73 (0.60-0.88)	.001	1.02 (0.84-1.24)	.84
Acute myocardial infarction	150 (1.18)	61 (1.44)	49 (1.16)	40 (0.95)	Erlotinib	0.85 (0.58-1.24)	.39	0.95 (0.65-1.39)	.81
					Afatinib	0.60 (0.40-0.90)	.01	0.81 (0.54-1.23)	.32
Ischemic stroke	862 (6.81)	310 (7.34)	285 (6.75)	267 (6.32)	Erlotinib	0.97 (0.83-1.14)	.73	1.02 (0.86-1.19)	.86
					Afatinib	0.79 (0.67-0.94)	.006	0.90 (0.76-1.06)	.22

Abbreviations: HR, hazard ratio; MACCEs, major adverse cardiac and cerebrovascular events; TKIs, tyrosine kinase inhibitors.

^a^ Model was adjusted for age, sex, stage, therapies used during (radiotherapy, operation, antiarrhythmia drugs, anticancer drugs, platinum analogues), cardiovascular medication (angiotensin-converting enzyme inhibitor/angiotensin receptor blocker, β-blocker, anti-platelet agents, anticoagulants, statins, digoxin, mineralocorticoid-receptor antagonists), comorbidities (coronary artery disease, peripheral artery disease, hypertension, diabetes, hyperlipidemia, valve disease, chronic obstructive lung disease, asthma, chronic kidney disease, end-stage kidney disease).

## Discussion

Although the use of TKIs is known to significantly improve survival in patients with *EGFR* sequence variation–positive NSCLC, their potential cardiotoxicity may threaten benefits in long-term mortality and morbidity.^15^ Notably, the use of TKIs in Asia is significantly higher than that in the Western countries owing to the high prevalence of the *EGFR* sequence variation in the Asian population.^9^ Therefore, it is crucial to understand and weigh the association of TKIs with both survival and cardiovascular outcomes. By analyzing this nationwide cohort, we found that patients with NSCLC receiving TKIs had a considerably lower likelihood of dying from any cause, particularly cancer-related causes, than patients who did not receive TKIs. Nevertheless, the TKI group had a higher incidence of MACCEs. Regarding the comparison of different types of TKIs, afatinib was associated with the most considerably reduced probability of death compared with erlotinib and gefitinib, but the HRs of MACCEs were similar between the groups. Altogether, these results highlighted that TKI use was associated with an increasing probability of MACCE, but its benefit in terms of cancer-related survival was not reduced. According to our study, cardiovascular problems should be closely monitored in patients taking TKIs. Early detection of cardiotoxicities and prompt treatment could increase the benefits of TKI therapy.^21,22^

Cancer therapeutics–related cardiotoxicity shows detrimental effects on the long-term outcomes in patients with cancer.^21,22^ Before TKIs, platinum-based chemotherapy was considered the standard treatment for patients with advanced NSCLC, but it has potential nephrotoxic, neurotoxic, and cardiotoxic effects.^23,24^ Moreover, AMI, autonomic dysfunction, and arrhythmia are some of the cardiovascular problems associated with platinum-based therapy.^24^ An increasing number of patients are now receiving TKI treatment based on the new research that shows TKIs improve survival in patients with *EGFR* sequence variations.^6,13^ Myocardial ischemia, HF, fatal arrhythmia, and hypertension have unfortunately been linked to TKI use.^14,25^ Given that patients with advanced NSCLC may consequently receive different anticancer therapies, we listed the details of all prescribed anticancer drugs in eTable 2 in Supplement 1. To identify the complex interaction between TKI use and other drugs, we found that the increasing HRs of MACCEs among those receiving TKI was independent of most comorbidities and the use of cardiovascular drugs. In contrast, TKI use was not associated with significantly increasing MACCEs in individuals receiving platinum-based therapy. This emphasizes the need for individuals receiving various anticancer medicines to be monitored individually for any potential cardiovascular issues.

We examined whether the outcomes of death and cardiovascular events differ in patients who received first- or second-generation TKIs, and our results showed that use of afatinib resulted in a significant reduction in HR of death, but the HRs of MACCEs among the 3 drugs were similar. According to the LUX-Lung 7 study, afatinib significantly prolonged progression-free survival compared with gefitinib in patients with *EGFR*-positive NSCLC.^26^ Although previous research has suggested that afatinib and osimertinib can contribute to the development of HF,^27^ whereas erlotinib and gefitinib have been associated with ischemic events, there is currently no comparison of the specific effects of TKIs on cardiovascular outcomes.^28,29^ To date, most studies have focused on osimertinib—the third-generation TKI. Despite a promising improvement in progression-free survival in the FLAURA study,^30^ osimertinib has been reported to be associated with increased complications of HF, atrial and ventricular fibrillation, AMI, and pericardial effusion compared with first-generation TKIs.^31,32^ The study did not compare osimertinib to other TKIs because it has yet included in NHIRD until 2020.

Although the underlying pathophysiology of TKI-related cardiotoxicity remains largely unknown, several possible mechanisms have been proposed.^16^ Mak et al^33^ reported that chronic erlotinib treatment contributed to hypomagnesemia, triggering substance P-receptor-mediated oxidative stress, resulting in cardiac dysfunction. Similarly, cisplatin—often used in combination therapy with TKIs—has been proposed to cause even more pronounced hypomagnesemia through magnesium wasting.^33,34^ Also, tyrosine kinases themselves have been reported having important functions in the transduction of extracellular signals that control the growth, differentiation, metabolism, migration, and death of cells.^6,15,35^ Stimulation of β1-adrenergic receptors induces EGFR transactivation to activate prosurvival signaling pathways in cardiomyocytes.^36,37^ Therefore, in conditions of high catecholamine secretion, erlotinib may block the cardioprotective signals through EGFR inhibition, resulting in the development of HF.^36,37^ TKIs may also cause endothelial dysfunction, coronary spasm, and thromboembolic events by increasing the activities of tissue factors in endothelial cells.^15,38^

### Limitations

Our study has limitations. First, owing to a relatively limited survival time in advanced NSCLC, patients may die before reaching cardiovascular end points. Alternatively, we adjusted for mortality as a competing risk and observed a sustained increase in MACCEs in the TKI group compared with the non-TKI group. However, the genetic differences between TKI and non-TKI groups may skew the results as TKIs were previously only recommended for people with *EGFR* sequence variations. Second, there is a lack of information on *EGFR* sequence variation–positive patients without TKI treatment. Additionally, osimertinib has been shown to have an increased threat of cardiotoxicity compared with first-generation TKIs.^31,32^ Osimertinib-related information is not included in the study as it is not yet available in NHIRD.

## Conclusions

In this cohort of patients with NSCLC, we found that TKI use was associated with a lower risk of cancer-related death but higher MACCEs than non-TKI use. Our results suggest a compelling argument for the need for ongoing surveillance for cardiovascular problems. In addition, we recommend that oncologists and cardiologists should work together to carefully monitor the potential cardiovascular toxic effects in patients treated with TKIs.

## Notes

Supplement 1. **eTable 1.** ICD-9 and ICD-10 Codes **eTable 2.** The Details of Anti-Arrhythmia and Anti-Cancer Drugs of Patients With Non-Small Cell Lung Cancers (NSCLC) Treated With or Without Tyrosine Kinase Inhibitors (TKIs) After Propensity Score Matching **eTable 3.** The Event Numbers of Patients With Non-Small Cell Lung Cancers (NSCLC) Treated With or Without Tyrosine Kinase Inhibitors (TKIs) **eTable 4.** The Crude and Adjusted Hazard Ratio (HR) of Patients With Non-Small Cell Lung Cancer (NSCLC) Treated With or Without TKIs **eTable 5.** The Reasons of Death of Patients With Non-Small Cell Lung Cancers (NSCLC) Treated With or Without Tyrosine Kinase Inhibitors (TKIs) **eTable 6.** The Baseline Characteristics of Patients With Non-Small Cell Lung Cancers (NSCLC) Treated With Different Tyrosine Kinase Inhibitors Including Erlotinib, Gefitinib and Afatinib After Propensity Score Matching **eTable 7.** The Crude and Adjusted Subdistribution Hazard Ratio (sHR) of Major Adverse Cardiac and Cerebrovascular Events (MACCEs) in Patients With Non-Small Cell Lung Cancers (NSCLC) Treated With Different Tyrosine Kinase Inhibitors **eTable 8.** The Reason of Death of Patients With Non-Small Cell Lung Cancers (NSCLC) Treated With Different Tyrosine Kinase Inhibitors **eFigure.** The Flowchart of Study Design

Supplement 2.
Data Sharing Statement
