# Supplementary material for: Assessment of Tyrosine Kinase Inhibitors and Survival and Cardiovascular Outcomes of Patients With Non–Small Cell Lung Cancer in Taiwan
Source: JAMA Netw Open. 2023 May 17;6(5):e2313824. doi: 10.1001/jamanetworkopen.2023.13824 (PMC10193184; doi:10.1001/jamanetworkopen.2023.13824)
Supplement: Supplement 1. — eTable 1. ICD-9 and ICD-10 Codes eTable 2. The Details of Anti-Arrhythmia and Anti-Cancer Drugs of Patients With Non-Small Cell Lung Cancers (NSCLC) Treated With or Without Tyrosine Kinase Inhibitors (TKIs) After Propensity Score Matching eTable 3. The Event Numbers of Patients With Non-Small Cell Lung Cancers (NSCLC) Treated With or Without Tyrosine Kinase Inhibitors (TKIs) eTable 4. The Crude and Adjusted Hazard Ratio (HR) of Patients With Non-Small Cell Lung Cancer (NSCLC) Treated With or Without TKIs eTable 5. The Reasons of Death of Patients With Non-Small Cell Lung Cancers (NSCLC) Treated With or Without Tyrosine Kinase Inhibitors (TKIs) eTable 6. The Baseline Characteristics of Patients With Non-Small Cell Lung Cancers (NSCLC) Treated With Different Tyrosine Kinase Inhibitors Including Erlotinib, Gefitinib and Afatinib After Propensity Score Matching eTable 7. The Crude and Adjusted Subdistribution Hazard Ratio (sHR) of Major Adverse Cardiac and Cerebrovascular Events (MACCEs) in Patients With Non-Small Cell Lung Cancers (NSCLC) Treated With Different Tyrosine Kinase Inhibitors eTable 8. The Reason of Death of Patients With Non-Small Cell Lung Cancers (NSCLC) Treated With Different Tyrosine Kinase Inhibitors eFigure. The Flowchart of Study Design [file jamanetwopen-e2313824-s001.pdf]

Supplemental Online Content

Chang WT, Lin HW, Chang TC, Lin SH, Li YH. Assessment of tyrosine kinase inhibitors and survival and cardiovascular outcomes of patients with non–small cell lung cancer in Taiwan. *JAMA Netw Open.* 2023;6(5):e2313824. doi:10.1001/jamanetworkopen.2023.13824

eTable 1. ICD-9 and ICD-10 Codes

eTable 2. The Details of Anti-Arrhythmia and Anti-Cancer Drugs of Patients With Non-Small Cell Lung Cancers (NSCLC) Treated With or Without Tyrosine Kinase Inhibitors (TKIs) After Propensity Score Matching

eTable 3. The Event Numbers of Patients With Non-Small Cell Lung Cancers (NSCLC) Treated With or Without Tyrosine Kinase Inhibitors (TKIs)

eTable 4. The Crude and Adjusted Hazard Ratio (HR) of Patients With Non-Small Cell Lung Cancer (NSCLC) Treated With or Without TKIs

eTable 5. The Reasons of Death of Patients With Non-Small Cell Lung Cancers (NSCLC) Treated With or Without Tyrosine Kinase Inhibitors (TKIs)

eTable 6. The Baseline Characteristics of Patients With Non-Small Cell Lung Cancers (NSCLC) Treated With Different Tyrosine Kinase Inhibitors Including Erlotinib, Gefitinib and Afatinib After Propensity Score Matching

eTable 7. The Crude and Adjusted Subdistribution Hazard Ratio (sHR) of Major Adverse Cardiac and Cerebrovascular Events (MACCEs) in Patients With Non-Small Cell Lung Cancers (NSCLC) Treated With Different Tyrosine Kinase Inhibitors

eTable 8. The Reason of Death of Patients With Non-Small Cell Lung Cancers (NSCLC) Treated With Different Tyrosine Kinase Inhibitors

eFigure. The Flowchart of Study Design

This supplemental material has been provided by the authors to give readers additional information about their work.

© 2023 Chang WT et al. *JAMA Network Open.*

eTable 1. ICD-9 and ICD-10 codes

Disease	ICD-9 Codes	ICD-10 Codes
Lung cancer	162	C34
Outcome		
Acute myocardial infarction	410	I21, I22, I23
Heart failure	428, 402.01, 402.11, 402.91, 404.01, 404.03, 404.11, 404.13, 404.91, 404.93, 785.51	I11.0, I13.0, I13.2, I42.0, I42.1, I42.2, I42.3, I42.4, I42.5, I42.6, I42.7, I42.8, I42.9, I43, I50.1, I50.20, I50.21, I50.22, I50.23, I50.30, I50.31, I50.32, I50.33, I50.40, I50.41, I50.42, I50.43, I50.9, R57.0
Ischemic stroke (including TIA)	433, 434, 435, 436	I63, I65, I66, I67.89, I67.84, G45.0, G45.1, G45.2, G45.8, G45.9, G46.0, G46.1, G46.2
Comorbidities		
Coronary artery disease	410, 411, 412, 413, 414	I20, I21, I22, I24, I25
Peripheral artery disease	440, 443, 444, 447.8, 447.9	I70.2-I70.9, I71, I73.9, I74.2, I74.3, I74.4, I74.5, I77.89, I77.9
Hypertension	401, 402, 403, 404, 405	I10, I11.0, I11.9, I12.0, I12.9, I13.0, I13.2, I13.11, I15, N26.2
Diabetes mellitus	250	E08, E09, E11, E13
Hyperlipidemia	272	E78
Valve disorders	394-397, 424.0, 424.1, 424.2, 424.3	I05, I06, I07, I08, I09, I34-I37
Chronic obstructive pulmonary disease	491, 492, 494, 495, 496	J41, J42, J43, J44, J47, J67
Asthma	493	J45
Atrial fibrillation	427.31, 427.32	I48
Chronic kidney disease	580-589, 403, 404, 585, V45.1, V56	I12, I13, N02, N03, N04, N05, N06, N07, N08, N11, N14, N17, N18, N19, N29, O10.2, O10.3, Q61, Z49,

© 2023 Chang WT et al. *JAMA Network Open.*

		Z99.2
ESRD	585	N18.6; Z99.2
Procedure Code		
Radiotherapy	V58.0	Z51.0
Operations	67023B (single lobectomy), 67042B(Bilobectomy), 67050B (Thoracoscopic Lobectomy)	

© 2023 Chang WT et al. *JAMA Network Open.*

eTable 2. The details of anti-arrhythmia and anti-cancer drugs of patients with non-small cell lung cancers (NSCLC) treated with or without tyrosine kinase inhibitors (TKIs) after propensity score matching.

	Total N=48258	TKI users N=24129	TKI non-users N=24129	ASMD
Anti-arrhythmia drugs				
Amiodarone	931 (1.93)	454 (1.88)	477 (1.98)	0.007
Dronedarone	33 (0.07)	23 (0.10)	10 (0.04)	0.02
Propafenone	206 (0.43)	107 (0.44)	99 (0.41)	0.005
Flecainide		11 (0.02)		0.02
Quinidine	22 (0.05)	11 (0.05)	11 (0.05)	0.000
Procainamide		0 (0.00)		
Anti-cancer drugs				
Gemcitabine	2361 (4.89)	1160 (4.81)	1201 (4.98)	0.008
Taxanes	2913 (6.04)	1565 (6.49)	1348 (5.59)	0.04
Docetaxel	2215 (4.59)	1249 (5.18)	966 (4.00)	0.06
Paclitaxel	764 (1.58)	353 (1.46)	411 (1.70)	0.02
Vinorelbine	3335 (6.91)	1620 (6.71)	1715 (7.11)	0.02
Pemetrexed	3520 (7.29)	2025 (8.39)	1495 (6.20)	0.09
Irinotecan		20 (0.041)		0.03
Avastin	10 (0.02)	3 (0.01)	7 (0.03)	0.01

Abbreviation as listed in Table 1

© 2023 Chang WT et al. *JAMA Network Open.*

eTable 3. The event numbers of patients with non-small cell lung cancers (NSCLC) treated with or without tyrosine kinase inhibitors (TKIs)

	Total<br>N=48258	TKI users<br>N=24129	TKI non-users (Ref)<br>N=24129
All cause death	37796 (78.32)	19338 (80.14)	18458 (76.50)
MACCEs	5278 (10.94)	2848 (11.80)	2430 (10.07)
Heart failure	2443 (5.06)	1262 (5.23)	1181 (4.89)
Acute myocardial infarction	511 (1.06)	283 (1.17)	228 (0.94)
Ischemic stroke	2822 (5.85)	1596 (6.61)	1226 (5.08)

Abbreviation as listed in Table 1

© 2023 Chang WT et al. *JAMA Network Open.*

eTable 4. The crude and adjusted hazard ratio (HR) of patients with non-small cell lung cancer (NSCLC) treated with or without TKIs

	Crude HR (95%CI)	*p* value	Adjusted HR (95%CI)	*p* value
All cause death	0.90 (0.88-0.92)	<0.001	0.76 (0.75-0.78)	<0.001
MACCEs	1.01 (0.95-1.06)	0.83	0.97 (0.92-1.03)	0.31
Heart failure	0.92 (0.85-0.99)	0.04	0.87 (0.80-0.94)	<0.001
Acute myocardial infarction	1.07 (0.90-1.27)	0.46	1.00 (0.84-1.20)	0.97
Ischemic stroke	1.12 (1.04-1.20)	0.004	1.10 (1.02-1.18)	0.02

TKI non-users are references.

Model was adjusted for age, sex, stage, therapies use during (radiotherapy, operation, anti-arrhythmia drugs, anti-cancer drugs, platinum analogues), CV medication (ACEI/ARB, beta blocker, anti-platelet agents, anti-coagulants, statins, digoxin, MRA) and comorbidities (coronary artery disease, peripheral artery disease, hypertension, diabetes mellitus, hyperlipidemia, valve disease, chronic obstructive lung disease, asthma, chronic kidney disease, ESRD).

HR= hazard ratio.

© 2023 Chang WT et al. *JAMA Network Open.*

eTable 5. The reasons of death of patients with non-small cell lung cancers (NSCLC) treated with or without tyrosine kinase inhibitors (TKIs)

	Total N=48258	TKI users N=24129	TKI non-users N=24129
Cancer death	33632 (69.69)	17357 (71.93)	16275 (67.45)
CV death	221 (0.46)	100 (0.41)	121 (0.50)
Others	3943 (8.17)	1881 (7.80)	2062 (8.55)

Abbreviation as listed in Table 1

© 2023 Chang WT et al. *JAMA Network Open.*

eTable 6. The baseline characteristics of patients with non-small cell lung cancers (NSCLC) treated with different tyrosine kinase inhibitors including Erlotinib, Gefitinib and Afatinib after propensity score matching.

	Total N=12666		Erlotinib user N=4222		Gefitinib user N=4222		Afatinib user N=4222		ASMD		
									E vs G	G vs A	E vs A
Age									0.15	0.42	0.27
Mean (SD)	67.34	(11.44)	67.75	(11.08)	69.50	(11.82)	64.77	(10.90)			
Median (IQR)	67.00	(16.00)	67.00	(16.00)	70.00	(18.00)	64.00	(16.00)			
Sex									0.11	0.14	0.03
Male	4759	(37.57)	1639	(38.82)	1418	(33.59)	1702	(40.31)			
Female	7907	(62.43)	2583	(61.18)	2804	(66.41)	2520	(59.69)			
Therapies for cancer											
Radiotherapy	284	(2.24)	114	(2.70)	92	(2.18)	78	(1.85)	0.03	0.02	0.06
Operations (lobectomy)	643	(5.08)	185	(4.38)	235	(5.57)	223	(5.28)	0.06	0.01	0.04
Anti-cancer drugs	1191	(9.40)	425	(10.07)	433	(10.26)	333	(7.89)	0.006	0.08	0.08
platinum analogues	467	(3.69)	151	(3.58)	156	(3.69)	160	(3.79)	0.006	0.005	0.01
CV medications											
ACEI/ARB	3656	(28.86)	1191	(28.21)	1301	(30.81)	1164	(27.57)	0.06	0.07	0.01
Beta blocker	2808	(22.17)	930	(22.03)	948	(22.45)	930	(22.03)	0.01	0.01	0.000
Anti-platelet agents	1899	(14.99)	633	(14.99)	689	(16.32)	577	(13.67)	0.04	0.07	0.04
Anti-coagulants	426	(3.36)	149	(3.53)	166	(3.93)	111	(2.63)	0.02	0.07	0.05
Statins	2257	(17.82)	722	(17.10)	799	(18.92)	736	(17.43)	0.05	0.04	0.009
Digoxin	128	(1.01)	46	(1.09)	42	(0.99)	40	(0.95)	0.009	0.005	0.01

© 2023 Chang WT et al. *JAMA Network Open.*

MRA	447	(3.53)	165	(3.91)	168	(3.98)	114	(2.70)	0.004	0.07	0.07
Anti-arrhythmia drugs	296	(2.34)	95	(2.25)	113	(2.68)	88	(2.08)	0.03	0.04	0.01
Comorbidities											
Coronary artery disease	1393	(11.00)	454	(10.75)	503	(11.91)	436	(10.33)	0.04	0.05	0.01
Peripheral artery disease	238	(1.88)	80	(1.89)	89	(2.11)	69	(1.63)	0.02	0.04	0.02
Hypertension	5610	(44.29)	1820	(43.11)	1999	(47.35)	1791	(42.42)	0.09	0.10	0.01
Diabetes mellitus	2633	(20.79)	884	(20.94)	941	(22.29)	808	(19.14)	0.03	0.08	0.05
Hyperlipidemia	3023	(23.87)	974	(23.07)	1055	(24.99)	994	(23.54)	0.05	0.03	0.01
Valve disease	367	(2.90)	127	(3.01)	136	(3.22)	104	(2.46)	0.01	0.05	0.03
Chronic obstructive lung disease	2201	(17.38)	761	(18.02)	737	(17.46)	703	(16.65)	0.01	0.02	0.04
Asthma	975	(7.70)	312	(7.39)	335	(7.39)	328	(7.77)	0.02	0.006	0.01
Chronic kidney disease	1100	(8.68)	372	(8.81)	411	(9.73)	317	(7.51)	0.03	0.08	0.05
ESRD	14	(0.11)	5	(0.12)					0.01	0.04	0.03

ASMD= absolute standardized mean difference; CV=cardiovascular; ACEI/ARB= angiotensin-converting enzyme inhibitor/Angiotensin Receptor Blocker; MRA= mineralocorticoid-receptor antagonists; ESRD=end-stage renal disease.

Abbreviation as listed in Table 1

© 2023 Chang WT et al. *JAMA Network Open.*

eTable 7. The crude and adjusted subdistribution hazard ratio (sHR) of major adverse cardiac and cerebrovascular events (MACCEs) in patients with non-small cell lung cancers (NSCLC) treated with different tyrosine kinase inhibitors.

		Crude sHR (95%CI)	*p* value	Adjusted sHR (95%CI)	*p* value
MACCEs	Erlotinib	0.91 (0.80-1.02)	0.11	0.98 (0.86-1.10)	0.70
	Afatinib	0.84 (0.74-0.95)	0.004	1.01 (0.89-1.15)	0.83
Heart failure	Erlotinib	0.88 (0.73-1.05)	0.17	0.99 (0.82-1.19)	0.88
	Afatinib	0.78 (0.64-0.94)	0.008	1.06 (0.87-1.29)	0.57
Acute myocardial infarction	Erlotinib	0.80 (0.55-1.17)	0.25	0.92 (0.63-1.34)	0.65
	Afatinib	0.65 (0.44-0.97)	0.04	0.86 (0.57-1.31)	0.50
Ischemic stroke	Erlotinib	0.92 (0.78-1.08)	0.30	0.96 (0.82-1.13)	0.64
	Afatinib	0.85 (0.73-1.01)	0.06	0.95 (0.80-1.12)	0.52

Model was adjusted for age, sex, stage, therapies use during (radiotherapy, operation, anti-arrhythmia drugs, anti-cancer drugs, platinum analogues), CV medication (ACEI/ARB, beta blocker, anti-platelet agents, anti-coagulants, statins, digoxin, MRA) and comorbidities (coronary artery disease, peripheral artery disease, hypertension, diabetes mellitus, hyperlipidemia, valve disease, chronic obstructive lung disease, asthma, chronic kidney disease, ESRD).
sHR = subdistribution hazard ratio.
Abbreviation as listed in Table 1

© 2023 Chang WT et al. *JAMA Network Open.*

eTable 8. The reason of death of patients with non-small cell lung cancers (NSCLC) treated with different tyrosine kinase inhibitors

	Total<br>N=12666	Erlotinib user<br>N=4222	Gefitinib user<br>N=4222	Afatinib user<br>N=4222
Cancer death	7889 (62.28)	2788 (66.04)	2620 (62.06)	2481 (58.76)
CV death	55 (0.43)	21 (0.50)	23 (0.54)	11 (0.26)
Others	800 (6.32)	256 (6.06)	273 (6.47)	271 (6.42)

Abbreviation as listed in Table 1

© 2023 Chang WT et al. *JAMA Network Open.*

eFigure 1. The flowchart of study design

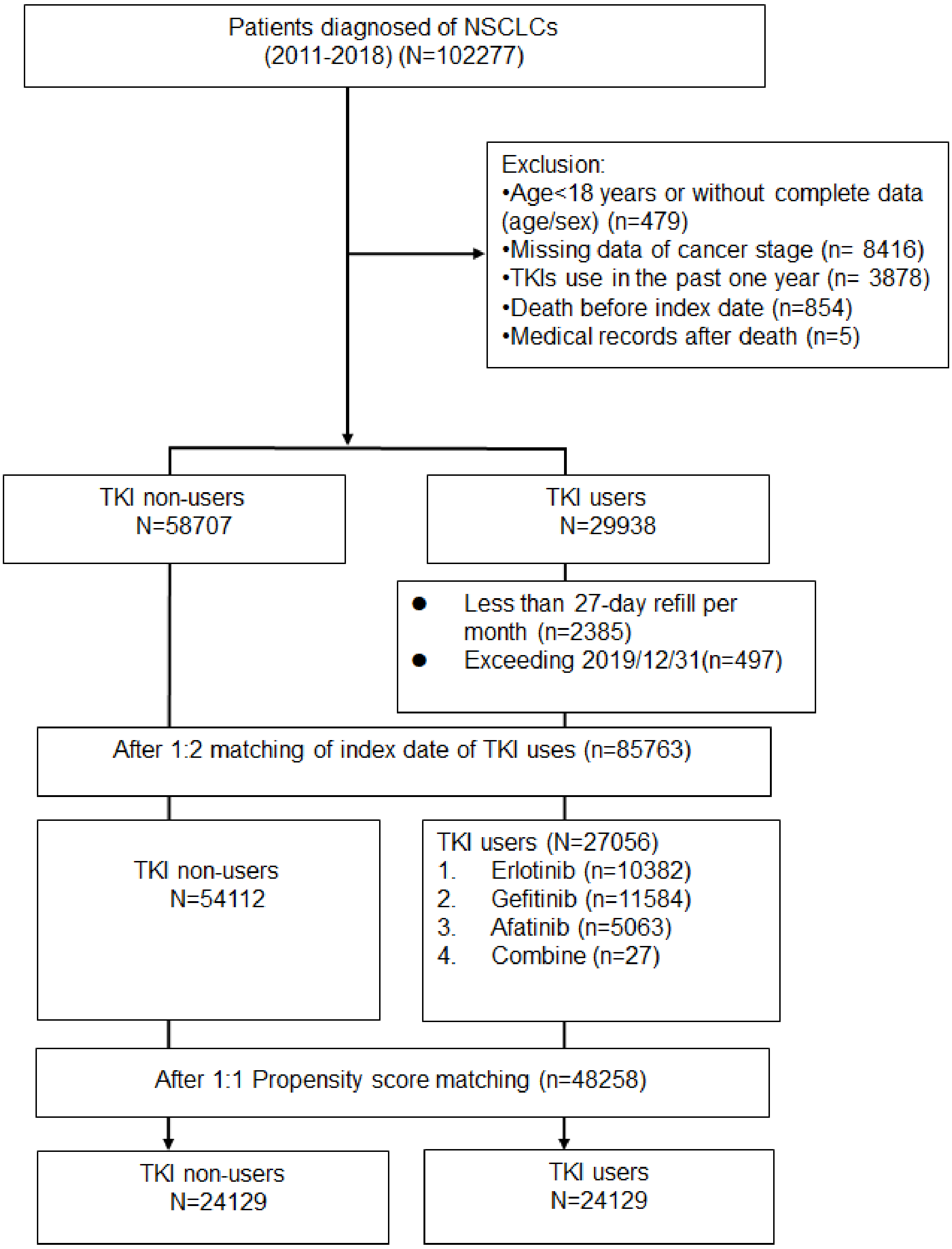

© 2023 Chang WT et al. *JAMA Network Open.*
