# Supplementary material for: Assessment of Tyrosine Kinase Inhibitors and Survival and Cardiovascular Outcomes of Patients With Non–Small Cell Lung Cancer in Taiwan
Source: JAMA Netw Open. 2023 May 17;6(5):e2313824. doi: 10.1001/jamanetworkopen.2023.13824 (PMC10193184; doi:10.1001/jamanetworkopen.2023.13824)
Supplement: Supplement 2. — Data Sharing Statement [file jamanetwopen-e2313824-s002.pdf]

Data Sharing Statement

Chang. Assessment of Tyrosine Kinase Inhibitors and Survival and Cardiovascular Outcomes of Patients With Non–Small Cell Lung Cancer in Taiwan. *JAMA Netw Open*. Published May 17, 2023. doi:10.1001/jamanetworkopen.2023.13824

Data
Data available: No

Additional Information
Explanation for why data not available: The original data belongs to NHIRD.
